# Nano-enabled plant genetic engineering for stress resilience: current advances and future directions

**DOI:** 10.3389/fpls.2026.1785807

**Published:** 2026-03-17

**Authors:** Salem M. AL-Amri

**Affiliations:** College of Science and Humanities, Department of Biology, Shaqra University, Dawadmi, Saudi Arabia

**Keywords:** gene delivery, plant genetic engineering, RNA interference, smart nanocarriers, stress resilience

## Abstract

Plant productivity and food security are increasingly threatened by abiotic and biotic stresses intensified by climate change. Plant genetic engineering offers powerful solutions to enhance stress resilience; however, conventional transformation approaches remain constrained by limited host range, low precision, tissue culture dependency, and regulatory concerns. In this context, nanotechnology has emerged as a transformative enabling platform for precise, efficient, and species-independent delivery of genetic cargo into plant systems. This review provides a comprehensive overview of recent advances in nano-enabled plant genetic engineering for stress resilience, highlighting the role of diverse nanocarriers, including carbon-based nanomaterials (NMs), metal and metal oxide nanoparticles (NPs), polymer-based nanocarriers, and metal–organic frameworks in delivering DNA, RNA interference constructs, and genome-editing components. These nanoplatforms overcome key biological barriers, protect nucleic acids from degradation, and enable controlled, targeted, and often transgene-free genetic modulation. Beyond delivery, many NMs exhibit intrinsic bioactivity, which can synergistically enhance plant stress tolerance through redox regulation, nutrient supplementation, and activation of stress-responsive pathways. The review also critically discusses regulatory and biosafety challenges associated with nano-enabled delivery systems, emphasizing the need for harmonized frameworks tailored to NMs-specific properties. Finally, future perspectives are outlined, focusing on biodegradable nanocarriers, organelle-specific targeting, and integration with CRISPR-based technologies to advance sustainable, precise, and climate-resilient crop improvement strategies.

## 1 Introduction

The rapid growth of the global population, together with the increasing impacts of climate change, poses serious challenges to food security and demands innovative strategies to improve crop resilience (Ijaz et al., 2023; Murtaza et al., 2025). Plants are continuously exposed to a wide range of abiotic (drought, salinity, extreme temperatures, heavy metals) and biotic stresses (pathogenic infections), which significantly constrain crop productivity (Riyazuddin et al., 2025; Wang et al., 2025). Plant genetic engineering offers substantial promise for the development of crops capable of coping with the growing pressures imposed by climate change, evolving pest resistance, and the demand for enhanced nutritional quality (Forner et al., 2023). During the Green Revolution, yield improvements were largely achieved through breeding-based genetic interventions that strengthened stress tolerance; however, these approaches were constrained by limited genetic precision (Squire et al., 2023; Steinwand and Ronald, 2020). Although widely used methods such as *Agrobacterium*-mediated transformation and biolistic gene delivery have proven effective, they are associated with several drawbacks, including high operational costs, technical complexity, and the risk of unintended genetic alterations caused by off-target effects (Ahmed et al., 2025; Cai et al., 2024). Consequently, there is a critical need to advance plant genetic engineering platforms that can enhance transformation efficiency while enabling precise and reliable trait integration.

Nanotechnology has emerged as a powerful enabling tool to overcome these challenges by offering innovative solutions for the targeted, efficient, and non-toxic delivery of genetic cargo into plant systems (Ahmar et al., 2021; Zarrabi et al., 2026). Nano-enabled platforms such as carbon-based NMs, metal and metal-oxide nanoparticles (NPs), polymeric nanocarriers serve as sophisticated delivery vehicles for genetic cargo, including plasmid DNA, RNA interference constructs, and CRISPR-Cas9 components, enabling targeted modifications that enhance stress resilience while minimizing off-target effects (Kumar et al., 2025; Sandhya et al., 2020; Shivashakarappa et al., 2025). NMs can function as both carriers and bioactive agents, providing dual benefits through nutrient supplementation, reactive oxygen species scavenging, and direct modulation of stress-responsive pathways (Oehlke et al., 2014; Zhu et al., 2026). This multifunctional approach represents a significant departure from conventional transformation methods, offering enhanced transformation efficiency, reduced plant tissue culture requirements, and the potential for species-independent delivery systems.

The integration of nanotechnology into plant genetic engineering has catalyzed development of novel methodologies that address long-standing limitations in plant transformation (Landry and Mitter, 2019). In a recent study, Cai et al. (2024) reported that mesoporous silica NPs (10 µg mL^−1^) enable non-toxic, topical siRNA delivery into mature plant leaves, achieving up to 98% transient and multi-gene silencing with strong potential for functional genomics and crop improvement. Similarly, Ben-Haim et al. (2024) demonstrated that casein protein NPs efficiently delivered plasmid DNA into intact tobacco (*Nicotiana benthamiana*) cells, enabling nuclear uptake and transient gene expression confirmed by fluorescence imaging and molecular analyses. Although previous review articles have been published on nano-enabled plant genetic engineering (Demirer et al., 2021; Squire et al., 2023), they primarily provide descriptive overviews of delivery platforms; in contrast, the present review offers a focused and critical evaluation of recent progress, explicitly analyzing mechanistic insights, stress-specific applications.

This study aims to critically evaluate recent advances in nano-enabled delivery systems for plant genetic engineering with a specific focus on enhancing stress resilience. It compares major NMs platforms in terms of delivery efficiency, cargo protection, cellular uptake mechanisms, and biosafety considerations across plant species. The review further identifies translational challenges, regulatory constraints, and future research directions to guide the rational design of scalable and sustainable nano-enabled crop improvement strategies.

## 2 Overview of plant genetic engineering for stress resilience

Plant genetic engineering has evolved into a sophisticated toolkit for enhancing crop resilience against environmental stresses through targeted molecular interventions (Verma et al., 2022). This approach involves the introduction, modification, or silencing of specific genes to improve plant performance under adverse conditions. Traditional transformation methods have relied primarily on *Agrobacterium*-mediated gene transfer and particle bombardment, which enable stable integration of foreign genes into plant genomes (Levengood et al., 2024; Su et al., 2023). However, the effectiveness of these conventional approaches is often limited by factors such as host range restrictions, random integration events that can disrupt endogenous genes, and the lengthy regeneration processes required to obtain transgenic plants. Additionally, the presence of integrated transgenes and selectable marker genes in the final product raises regulatory concerns and public acceptance challenges in many agricultural systems (Abdul Aziz et al., 2022; Ahmad et al., 2021; Su et al., 2023). The advent of precision genome editing technologies, particularly CRISPR/Cas systems, has revolutionized the landscape of plant genetic engineering for stress resilience by enabling targeted modifications without necessarily introducing foreign DNA (Wada et al., 2022). This technology allows researchers to precisely delete, insert, or modify specific DNA sequences associated with stress responses, offering unprecedented control over trait development (Ali et al., 2023; Khan et al., 2021). For instance, genome editing has been applied to knock out negative regulators of stress tolerance pathways, fine-tune the expression of stress-responsive genes, and engineer multiple traits simultaneously through multiplex editing strategies (Fernie and Bulut, 2025; Karavolias et al., 2021). Moreover, RNA interference and other RNA-based approaches have emerged as powerful tools for post-transcriptional gene silencing, allowing transient or heritable suppression of genes that negatively impact stress tolerance (Hada et al., 2021; Shikha et al., 2025). Despite these advances, significant challenges remain in delivering these molecular tools efficiently across diverse plant species, achieving stable and heritable modifications in recalcitrant crops, and ensuring precise spatial and temporal control of gene expression to maximize stress resilience without compromising plant development or yield potential under normal growing conditions (Demirer et al., 2021; Yan et al., 2022). Collectively, these limitations of conventional transformation approaches underscore the need for innovative delivery strategies capable of overcoming species barriers, improving precision, and enabling efficient, non-integrative genetic modulation in plants.

## 3 Nano-enabled platforms for gene delivery

**Figure 1:**
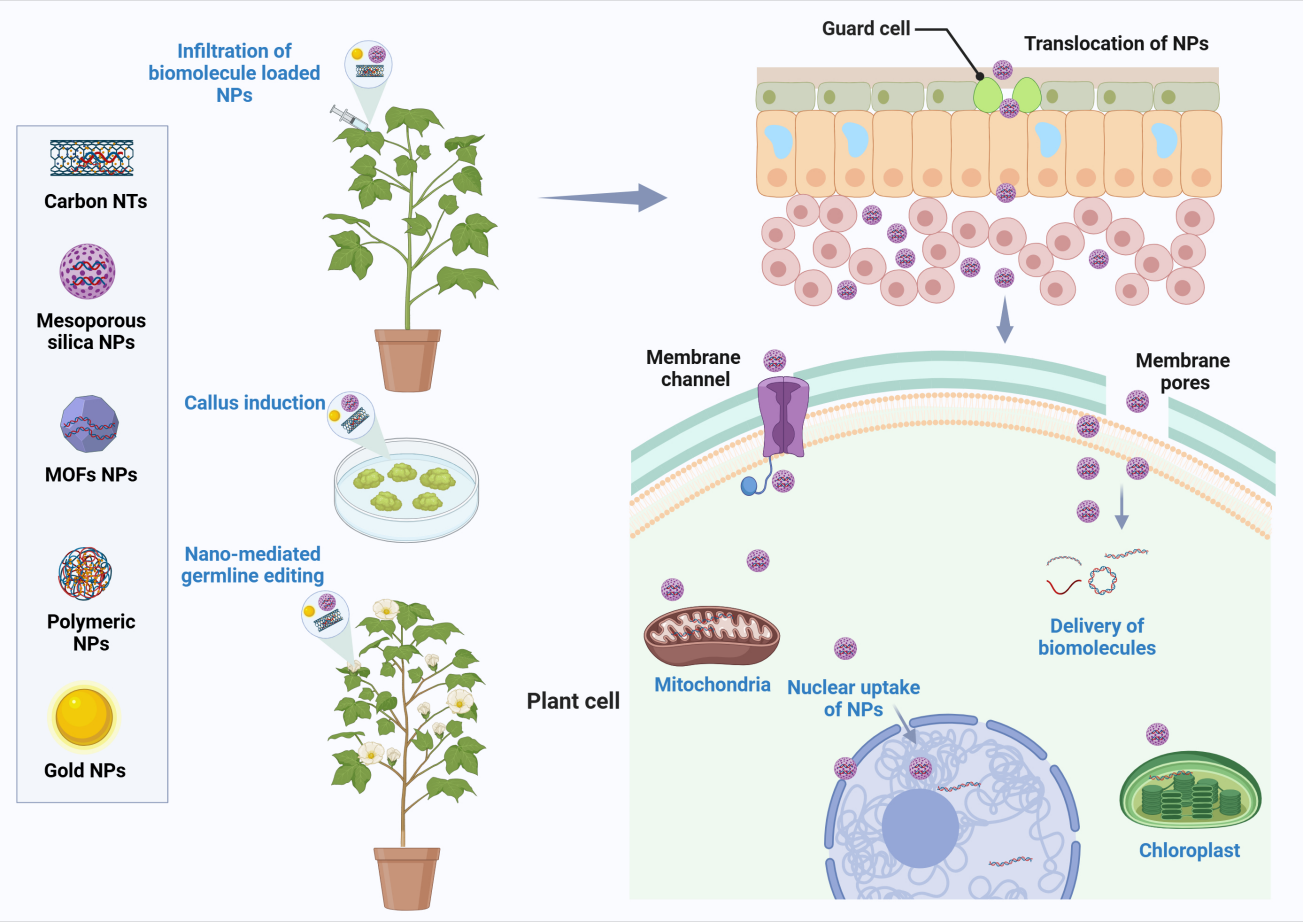
Schematic representation of nanomaterial-mediated delivery mechanisms for plant genetic engineering. The figure illustrates various nanocarrier platforms including carbon nanotubes, mesoporous silica nanoparticles, metal-organic frameworks, polymeric nanoparticles, and gold nanoparticles used for delivering genetic cargo into plant systems. These nano-enabled delivery systems can be applied through infiltration of biomolecule-loaded nanoparticles, callus induction, or nano-mediated germline editing. Nanoparticles translocate across guard cells and epidermal layers, enter plant cells through membrane channels and pores, and facilitate targeted delivery of biomolecules to specific subcellular compartments including the nucleus, mitochondria, and chloroplasts, enabling precise genetic modifications for enhanced stress resilience. Figure was created using Biorender.

**Figure 2:**
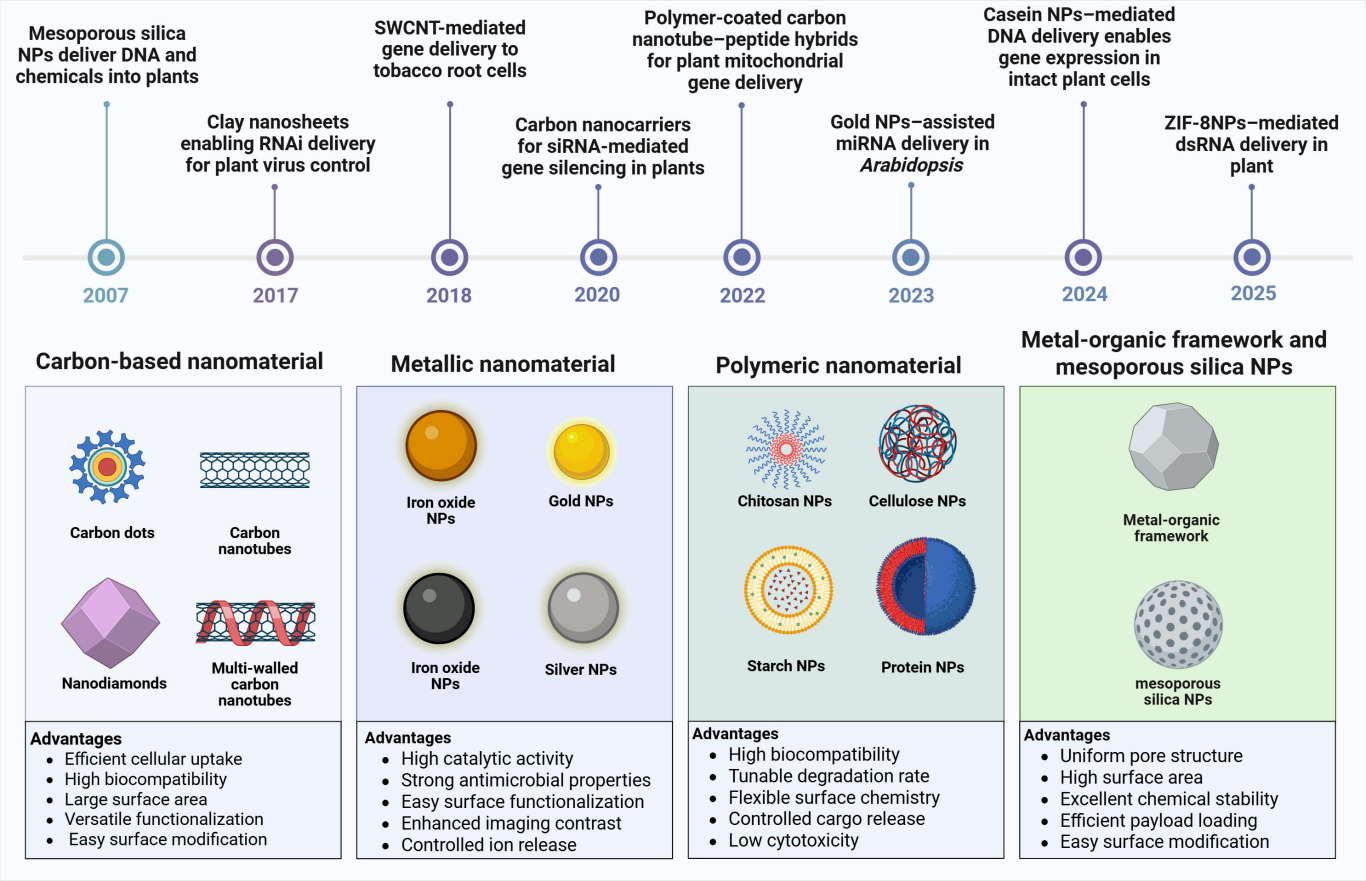
Timeline and classification of nano-enabled delivery platforms for plant genetic engineering. The upper panel presents a chronological timeline of key milestones in nanomaterial-mediated plant genetic engineering, beginning with mesoporous silica gene delivery into plants (Torney et al., 2007), followed by advances in clay nanosheets-enabled RNAi delivery for plant virus control (Mitter et al., 2017), single-walled carbon nanotube (SWCNT) functionalized with Arg and peptides-mediated gene delivery to tobacco root cells (Golestanipour et al., 2018), carbon nanocarriers for siRNA-mediated gene silencing in plants (Demirer et al., 2020), polymer-coated carbon nanotube–peptide hybrids for plant mitochondrial gene delivery (Law et al., 2022), gold NPs-assisted miRNA delivery in Arabidopsis (Zhang et al., 2023), casein nanoparticles (NPs)-mediated DNA delivery enabling gene expression in intact plant cells (Ben-Haim et al., 2024), and zinc imidazolate framework-8 nanoparticles (ZIF-8NPs)-mediated double-stranded RNA (dsRNA) delivery in plants against insect genes (Du et al., 2025). Figure was created using Biorender.

Nano-enabled platforms have emerged as transformative tools that address the fundamental challenges associated with delivering genetic materials into plant cells (Kodaparthi et al., 2025). Unlike conventional transformation methods that require extensive tissue culture and regeneration protocols, nanocarrier-based delivery systems can penetrate intact plant tissues through size-dependent mechanisms, surface charge interactions, and active transport processes as shown in **Figure 1** (Niazian et al., 2021). These nanoscale vehicles, typically ranging from 10 to 500 nanometers in diameter, can be engineered with specific physicochemical properties such as surface functionalization (**Figure 2**), charge optimization, and responsive release mechanisms to enhance cellular uptake and protect genetic cargo from enzymatic degradation (Liu et al., 2025; Mendes et al., 2022). The versatility of nano-enabled platforms extends beyond simple cargo delivery, as they can be designed to achieve organelle-specific targeting, controlled and sustained release of bioactive molecules, and multiplexed delivery of different genetic components simultaneously (Rani et al., 2025; Singh et al., 2024). Furthermore, these systems offer the potential for species-independent transformation, reduced dependence on tissue culture, and the achievement of transgene-free genome editing through transient delivery of CRISPR/Cas ribonucleoproteins, addressing both technical limitations and regulatory concerns associated with genetically modified organisms (Demirer et al., 2021).

### 3.1 Carbon-based nanomaterials

Carbon-based NMs, including carbon nanotubes (CNTs), graphene, graphene oxide, and carbon dots, have gained significant attention as nanocarriers for plant genetic engineering due to their exceptional physicochemical properties (**Figure 2**). These materials possess high surface area, mechanical robustness, and tunable surface functionalities that enable efficient adsorption and protection of nucleic acids through hydrophobic interactions, or electrostatic binding (Chandrasekaran et al., 2020; Gyawali et al., 2024; Xu et al., 2023). Importantly, their nanoscale dimensions and needle-like or sheet-like morphology facilitate passive penetration through the plant cell wall and plasma membrane, bypassing the need for physical or biological transformation agents (Ashfaq et al., 2025; Santana et al., 2022). Beyond their delivery capabilities, carbon-based NMs offer additional advantages that are particularly relevant for stress resilience engineering. Several studies have shown that these NMs can modulate plant physiological processes by enhancing photosynthetic efficiency, regulating reactive oxygen species homeostasis, and activating stress-responsive signaling pathways (Guirguis et al., 2023; Safdar et al., 2022).

In a previous study, carbon nanotube (2 mg L^-1^) based siRNA delivery achieves efficient gene silencing in *N. benthamiana* plant cells by protecting RNA from degradation and ensuring effective intracellular transport, offering a versatile alternative to conventional DNA vector methods for diverse plant biotechnology applications (Demirer et al., 2020). Gyawali et al. (2024) demonstrated that polyethylenimine-functionalized graphene quantum dots efficiently delivered dsRNA targeting *MGV1* and *RAS1*, enhancing gene silencing and significantly reducing *Fusarium graminearum* growth and wheat spike infection compared with naked dsRNA applications potentially. This study reveals the promising potential of graphene quantum dots as carriers for dsRNA-based fungal disease management in wheat and other crops. Similarly, Wang et al. (2020) reported that polyethyleneimine coated carbon dot nanocomposites enable efficient, DNase-protective DNA delivery and strong transient gene expression in intact crops, including rice (*Oryza sativa* L. japonica), wheat (*Triticum aestivum* L.), and mung bean (*Vigna radiata* L.), overcoming cell wall barriers and inducing traits. These results demonstrate a highly efficient DNA delivery system that enables immediate gene expression, offering a rapid and practical tool for gene engineering in plant biology research. Lin et al. (2023) revealed that carbon quantum dots translocate systemically in *Arabidopsis thaliana*, interact with microRNAs, alter epigenetic regulation of Argonaute genes, and induce heritable transcriptional changes, revealing carbon quantum dots-mediated modulation of plant gene regulation. In another study, functionalized carbon quantum dots with balanced amino/hydroxyl ratios enable efficient dsRNA delivery to rice brown planthoppers via plant vascular systems, increasing pest control efficiency from 20% to 80% through systemic plant-mediated transport (Tang et al., 2025). Collectively, these attributes position carbon-based NMs as powerful platforms for species-independent, transgene-free plant genetic engineering aimed at improving tolerance to abiotic and biotic stresses (**Table 1**).

**Table 1:** Nano-enabled delivery of genetic materials for sustainable agriculture.

Plants	Nanocarriers	Genetic materials	Delivery methods	Advantages	References
*Nicotiana benthamiana*	CDs	siRNA	Low-pressure spray	Small, efficient gene silencing without cell wall penetration barriers or toxicity.	(Yu et al., 2024)
*Nicotiana benthamiana*	GONs	siRNA	Leaf infiltration	Transient gene silencing avoiding viral vectors or Agrobacterium-mediated transformation methods.	(Li et al., 2022)
*Triticum aestivum*	polyethyleneimine functionalized CDs	DNA	Spray method	Non-integrating gene delivery avoiding cell wall barriers and host limitations.	(Wang et al., 2020)
*Arabidopsis thaliana*	CDs	DNA	Syringe infiltration	Efficient systemic transport enabling epigenetic gene regulation without chemical pesticide application.	(Lin et al., 2023)
*Oryza sativa*	CDs	dsRNA	Spray method	Efficient systemic pest control achieving 80% protection without chemical pesticide dependency.	(Tang et al., 2025)
*Zea maize*	Fe_3_O_4_ MNPs	DNA	Pollen magnetofection incubation	Culture-free, genotype-independent maize transformation avoiding tissue culture through pollen transfection method.	(Wang et al., 2022)
*Arabidopsis thaliana*	AuNPs	siRNA	Leaf infiltration	Simple, effective, quick gene silencing in plants without cytotoxicity concerns.	(Lei et al., 2020)
*Nicotiana benthamiana*	polyethylenimine-functionalized AuNPs	siRNA	Leaf infiltration	Efficient, non-toxic gene silencing without species limitations or genome integration.	(Zhang et al., 2021)
*Catharanthus roseus*	Superparamagnetic FeONPs	DNA	Vacuum infiltration	Species-independent, efficient gene delivery avoiding specialized equipment or tissue culture requirements.	(Abedini et al., 2025)
*Arabidopsis thaliana*	polyethyleneimine functionalized AuNPs	siRNA	Leaf infiltration	Environmentally friendly, efficient bacterial disease resistance without chemical pesticide dependency concerns.	(Qi et al., 2024)
*Nicotiana* *benthamiana*	Chitosan NPs	dsRNA	Syringe infiltration	Cost-effective, long-lasting viral protection without expensive RNA production requirements needed.	(Xu et al., 2023)
*Nicotiana benthamiana*	Casein NPs	DNA	Leaf infiltration	Efficient, non-toxic gene delivery enabling successful plant cell transfection and expression.	(Ben-Haim et al., 2024)
*Nilaparvata lugens*	ZIF-8	dsRNA	microinjection	Efficient, stable pest control targeting lethal genes without chemical pesticide dependency.	(Du et al., 2025)
*Nicotiana benthamiana* and *Arabidopsis thaliana*	ZIF-8	DNA/RNA	Leaf infiltration	Efficient, biocompatible gene delivery overcoming plant cell wall barriers without toxicity.	(Yu et al., 2024)

### 3.2 Metal and metal oxide nanoparticles

Metal and metal oxide NPs, such as gold, silver, iron oxide, and zinc oxide have been extensively explored as gene delivery vehicles in plant systems due to their structural stability (**Figure 2**), controlled size distribution, and ease of surface functionalization (Shivashakarappa et al., 2025; Zhang et al., 2022; Zhao et al., 2017). These NPs can bind genetic cargo through electrostatic interactions, covalent linkers, or polymer coatings, thereby protecting nucleic acids from degradation during transport and delivery (Gogoi et al., 2025; Zhi et al., 2022). In addition to serving as delivery platforms, many metal and metal oxide NPs exhibit intrinsic bioactivity that can synergistically enhance plant stress tolerance. For instance, iron and zinc oxide NPs can contribute to micronutrient supplementation (Ali et al., 2024; Manzoor et al., 2023), while copper NPs have been shown to improve resistance against drought and pathogen invasion (Kashyap et al., 2023). Furthermore, metal-based nanocarriers can influence redox balance and stress-responsive gene expression, providing dual functionality as both genetic delivery agents and stress modulators (Chauhan et al., 2023; Mazaheri-Tirani et al., 2022; Zia-ur-Rehman et al., 2023).

For example, Abedini et al. (2025) demonstrated that green-synthesized superparamagnetic iron oxide NPs enable simplified nanocarrier-based genetic transformation, achieving efficient DNA delivery and transient gene expression in *Catharanthus roseus*, highlighting a sustainable and species-flexible approach for plant genetic engineering. In another recent study, Qi et al. (2024) proved that polyethyleneimine-functionalized gold NPs enable traceable siRNA delivery and effective gene silencing in *Arabidopsis thaliana* plants, enhancing resistance to bacterial pathogens *Pseudomonas syringae*, while reducing oxidative stress. This research reveals how gold nanoparticle-mediated RNAi can enhance plant disease resistance, showcasing nanobiotechnology’s potential to protect agricultural systems (**Table 1**). Similarly, Zhang et al. (2023) confirmed that gold nanoparticle–mediated artificial miRNA delivery efficiently silenced *ATG6* in *Arabidopsis thaliana*, revealing a rapid and persistent transient loss-of-function system. ATG6 silencing reduced resistance against *Pseudomonas syringae* pv. *maculicola*, highlighting autophagy involvement in plant immunity.

### 3.3 Polymer-based nanocarriers

Polymer-based nanocarriers represent a highly versatile and biocompatible class of delivery systems for plant genetic engineering, encompassing natural polymers such as chitosan, alginate, and cellulose, as well as synthetic polymers as shown in **Figure 2** (Proenca et al., 2022; Tiwari et al., 2024; Yan et al., 2022). These materials can be engineered to form NPs with tunable size, surface charge, and degradation profiles, allowing efficient complexation and protection of nucleic acids (Ahmed et al., 2025; Silva et al., 2010). Polymer-based nanocarriers facilitate cellular uptake through electrostatic interactions with the negatively charged plant cell wall and membrane, while their flexible structures promote intracellular trafficking and controlled release of genetic cargo (Parkinson et al., 2022; Vinzant et al., 2025). A key advantage of polymer-based nanocarriers lies in their biodegradability and low phytotoxicity, which address biosafety and environmental concerns associated with NMs use in agriculture. Recent advances have demonstrated that polymeric and protein-based NPs can successfully deliver plasmid DNA and siRNA into intact plant cells. For example, Ben-Haim et al. (2024) demonstrated that casein NPs efficiently delivered DNA into intact *Nicotiana benthamiana* cells, enabling nuclear uptake and gene expression. This protein-based nanocarrier provides a tunable, traceable, and biocompatible platform for plant genetic delivery (**Table 1**). In another study, chitosan quaternary ammonium salt, amine-functionalized silica NPs, and carbon quantum dots efficiently delivered double-stranded RNA into *Nicotiana benthamiana*, enabling sustained RNA interference–mediated gene silencing and enhanced resistance against cucumber mosaic virus (Xu et al., 2023). In another study, Law et al. (2022) revealed that peptide-modified carbon nanotube–polymer hybrids enable highly efficient DNA delivery into *Arabidopsis thaliana* plant mitochondria, overcoming cell wall and organelle barriers. Mitochondrial gene integration enhanced plant growth, highlighting a powerful platform for metabolic engineering and organelle-targeted plant bioengineering. These features make polymer-based nanocarriers promising candidates for sustainable, scalable, and regulatory-friendly nano-enabled plant genetic engineering strategies.

### 3.4 Metal-organic framework-based nanocarriers

Metal-organic frameworks (MOFs) represent an emerging and highly promising class of nanocarriers for plant genetic engineering due to their unique hybrid structure (**Table 1**), consisting of metal ions or clusters coordinated with organic ligands to form highly porous and tunable frameworks (Huang et al., 2023; Yu et al., 2024). The exceptionally high surface area, adjustable pore size, and modular chemistry of MOFs enable efficient encapsulation, protection, and controlled release of diverse genetic cargos (He et al., 2023). MOFs can be rationally engineered to respond to specific intracellular stimuli such as pH, redox conditions, or enzymatic activity, allowing spatiotemporally controlled gene release within plant cells (Khan et al., 2025; Yang et al., 2021). In addition, surface functionalization of MOFs with polymers, peptides, or targeting ligands enhances their stability in planta, promotes cell wall penetration, and facilitates subcellular or organelle-specific delivery (Bindra et al., 2023; Wang et al., 2023). The application of MOFs in plant genetic engineering is still in its early stages, yet preliminary studies demonstrate their potential for efficient delivery of genetic cargo components across plant cell barriers. For example, Yu et al. (2024) demonstrated that zeolitic imidazolate framework-8 (ZIF-8) NPs (below 20 nm) effectively deliver DNA and RNA into intact *Nicotiana benthamiana* leaves and *Arabidopsis thaliana* roots while protecting RNA from enzymatic degradation and achieving successful gene silencing through siRNA delivery, highlighting MOFs as promising simplified platforms for advancing transgene-free crop improvement. In another study, Du et al. (2025) utilized ZIF-8 NPs to deliver dsRNA targeting *NlCYP303A1*, a molting-related gene in *Nilaparvata lugens*, achieving dramatically enhanced and persistent gene silencing compared to naked dsRNA in both feeding and rice-seedling dip experiments, demonstrating that ZIF-8 effectively protects dsRNA from degradation while improving interference efficiency for innovative agricultural pest management strategies (**Table 1**). Collectively, MOF-based nanocarriers provide a versatile and smart delivery platform with significant potential to advance precision plant genetic engineering for enhanced abiotic and biotic stress resilience.

## 4 Regulatory and biosafety challenges for nano-enabled delivery systems

The deployment of nano-enabled delivery systems in plant genetic engineering faces significant regulatory challenges due to the absence of harmonized, NMs-specific frameworks in agricultural biotechnology (Kumar et al., 2025; Liu et al., 2025). Existing regulations are largely designed for conventional genetically modified organisms or agrochemicals and often fail to adequately account for the unique physicochemical properties of NMs, such as size-dependent behavior, surface functionalization, persistence, and transformation in complex environmental matrices (Lombi et al., 2019). Key regulatory concerns include uncertainties surrounding nanoparticle fate, transport, and accumulation in soil-plant systems, as well as potential impacts on non-target organisms, soil microbiota, and food safety (Wu et al., 2025). In addition, distinguishing between transgenic, transgene-free, and transient nano-enabled gene delivery outcomes remains a regulatory gray area, particularly for CRISPR/Cas and RNA-based approaches that do not result in stable DNA integration (Squire et al., 2023). The lack of standardized protocols for NMs characterization, exposure assessment, and long-term ecotoxicological evaluation further complicates risk assessment and approval processes (El-Kady et al., 2023). Moreover, regulatory inconsistencies across regions hinder technology translation and commercialization, creating barriers for large-scale agricultural adoption. Concretely, future regulatory frameworks should mandate standardized physicochemical characterization (e.g., size distribution, surface charge, dissolution rate), tiered environmental risk assessment integrating life-cycle analysis, and crop exposure-specific toxicity thresholds supported by multi-season field data. Addressing these challenges will require the development of science-based, adaptive regulatory frameworks that integrate nanotechnology-specific risk assessment tools, life cycle analyses, and clear classification criteria to ensure the safe, transparent, and responsible use of nano-enabled delivery systems in sustainable crop improvement (Squire et al., 2023).

## 5 Concluding remarks and future outlook

Nano-enabled delivery systems are emerging as powerful tools for plant genetic engineering. They offer better control over transporting and protecting nucleic acids, proteins, and bioactive molecules, helping these substances cross plant barriers more effectively than traditional methods. Advances in nanocarrier design, including carbon-based NMs, polymeric NPs, inorganic systems, and metal-organic frameworks have significantly improved delivery efficiency, cargo stability, and spatiotemporal regulation of gene expression without reliance on traditional transgenic approaches. Collectively, these innovations position nanotechnology as a powerful platform to accelerate crop improvement, enhance stress resilience, and enable precise modulation of plant physiological and molecular responses under changing environmental conditions.

Looking ahead, future research should prioritize the rational design of biodegradable plant-compatible nanocomposites and nanohybrids with tunable physicochemical properties and organelle-specific targeting capabilities. Integrating nanotechnology with emerging tools such as CRISPR-based genome editing, RNA interference, and artificial intelligence-guided material optimization will further expand the precision and scalability of plant engineering strategies. In particular, although nano-enabled CRISPR platforms are increasingly proposed for DNA-free genome editing, current evidence shows variable efficiencies between ribonucleoprotein (RNP) and plasmid delivery, challenges in maintaining RNP stability and activity during cellular transport, and limited systematic evaluation of off-target editing frequencies following nano-mediated delivery. Bridging laboratory-scale successes with real-world agricultural applications will require interdisciplinary collaboration among plant biologists, materials scientists, and regulatory bodies, ultimately enabling nano-enabled technologies to contribute meaningfully to sustainable and climate-resilient agriculture.
